# Staff Conditions in Hospitals

**Published:** 1919-09-27

**Authors:** 

**Affiliations:** O. P. Club.


*To the Editor of* THE HOSPITAL.

### Staff Conditions in Hospitals.

*To the Editor of* THE HOSPITAL.

SIR,—Having a life-long practical experience within the walls of large nursing institutions I am keenly interested in THE HOSPITAL and the particulars therein set forth. I note with pleasure your demand for an independent inquiry by competent persons into the conditions under which many nurses serve in various institutions—in my opinion such inquiry is necessary and would prove very salutary. In your last issue you make protest against the treatment of two unwilling witnesses (nurses) in respect to defective food conditions in an infirmary. In your correspondence particulars are given as to the conditions obtaining in a large hospital—food supplies and service, sanitation of rooms, taxation of private means of the nurse to augment food, also providing for ultra polishing of wards, appliances, and laundry. My experience was that insufficient care was bestowed upon the night staffs' food supplies and service, despite the fact of setting up suitable appliances. Also that nurses do not give evidence of defects because of the well-grounded fear of victimisation, and frequently because the end of the training sees the end of the worry which follows complaints.

As regards infirmaries, certain inspectors have access and are principally concerned about the wellbeing of patients, the wellbeing of the staff necessarily secondary. I am not aware if any competent inquiry is made at the various hospitals as to staff conditions, but surely such inquiry would be very wholesome.—I am, Sir,

Your Obedient Servant,

